# Mechanism of circADD2 as ceRNA in Childhood Acute Lymphoblastic Leukemia

**DOI:** 10.3389/fcell.2021.639910

**Published:** 2021-05-13

**Authors:** Yuting Zhu, Xiaopeng Ma, Heng Zhang, Yijun Wu, Meiyun Kang, Yongjun Fang, Yao Xue

**Affiliations:** ^1^Department of Hematology and Oncology, Children’s Hospital of Nanjing Medical University, Nanjing, China; ^2^Key Laboratory of Hematology, Nanjing Medical University, Nanjing, China

**Keywords:** circADD2, ceRNA, miR-149-5p, childhood ALL, AKT2

## Abstract

**Background:** Acute lymphocytic leukemia (ALL) is the most common malignant tumor in children. Increasing evidence suggests that circular RNAs (circRNAs) play critical regulatory roles in tumor biology. However, the expression patterns and roles of circRNAs in childhood acute lymphoblastic leukemia (ALL) remain largely unknown.

**Methods:** circADD2 was selected by microarray assay and confirmed by qRT-PCR; *in vitro* effects of circADD2 were determined by CCK-8 and flow cytometry; while mice subcutaneous tumor model was designed for *in vivo* analysis. RNA immunoprecipitation and dual-luciferase assay were applied for mechanistic study. Protein levels were examined by Western blot assay.

**Results:** circADD2 was down-regulated in ALL tissues and cell lines. Overexpression of circADD2 inhibited cell proliferation and promoted apoptosis both *in vitro* and *in vivo*. Briefly, circADD2 could directly sponge miR-149-5p, and the level of AKT2, a target gene of miR-149-5p, was downregulated by circADD2.

**Conclusion:** circADD2, as a tumor suppressor in ALL, can sponge miR-149-5p, and may serve as a potential biomarker for the diagnosis or treatment of ALL.

## Introduction

Acute lymphoblastic leukemia (ALL), a clonal dysplastic disease originating from the bone marrow where B-line or T-line lymphocytes are generated, is commonly found in children (Iacobucci and Mullighan, 2017; Teachey and Pui, 2019). The prognosis of childhood ALL has been significantly improved. However, relapses even deaths still appear due to treatment failure (Inaba et al., 2013; Hunger and Mullighan, 2015). Therefore, the pathogenesis of ALL needs further exploration before the establishment of new treatment options.

Circular RNAs (circRNAs) are a class of endogenous non-coding RNAs, characterized by a covalent closed-loop structure without neither a 5′ cap nor a 3′ Poly A tail (Meng et al., 2017; Yu et al., 2019). Unlike linear RNAs, circRNAs demonstrate remarkable stability, high abundance, evolutionary conservation, and tissue-specific expression (Aufiero et al., 2019). New RNA sequencing (RNA-seq) technology has discovered more functional circRNAs (Szabo and Salzman, 2016). In multiple cancers, circRNAs regulate important cellular processes, including proliferation, invasion and metastasis (Wang et al., 2018; Zeng et al., 2018). However, the mechanisms of circRNAs in ALL remain unclear.

MicroRNA-149-5p (miR-149-5p) is dysregulated in multiple tumors (Ye et al., 2019; Zhang et al., 2019). Studies have shown that miR-149-5p acts an oncogene in leukemia through facilitating the proliferation and apoptosis (Tian and Yan, 2016). Bearing miRNA binding sites, some circRNAs can serve as “miRNA sponges” (Chen X. et al., 2019; Shang et al., 2019). However, the interplay between circRNAs and related miRNAs in childhood ALL has not been elucidated.

In the present study, we first identified that circADD2 is derived from the ADD2 gene, downregulated in bone marrow and cell lines of childhood ALL, and involved in the progression of ALL by sponging miR-149-5p. Our findings provide a potential clinical marker for childhood ALL.

## Materials and Methods

### Clinical Samples

The study was approved by the Ethics Committee of Nanjing Medical University and all patients received written consent from their parents. Thirty bone marrow samples were obtained from newly diagnosed pediatric ALL patients receiving therapy at Children’s Hospital of Nanjing Medical University (Nanjing, China) during 2018 and 2019. The control samples were obtained from children without malignant diseases.

### Cell Lines

Jurkat, 6T-CEM (T-ALL lines), Nalm-6 (B-ALL lines), and 293T cells were purchased from the Shanghai Institute of Cell Biology, Chinese Academy of Sciences (Shanghai, China). The Jurkat,6T-CEM, and Nalm-6 cells were cultured in RPMI 1640 (Gibco, Carlsbad, CA, United States), 293T cells were cultured with DMEM (Gibco, Carlsbad, CA, United States) containing 10% fetal bovine serum and 1% penicillin and streptomycin. All these cell lines were maintained at 37°C with 5% CO_2_ in a humidified incubator.

### Cell Transfection

For analyze circRNA overexpression, pcDNA-based circADD2 overexpression vector and pcDNA vector were synthesized by Genepharma (Shanghai, China). Stable transfection in ALL cells was performed according to manufacturer agreement. MiR-149-5p mimics (or miR-149-5p inhibitor) and NC mimics (or NC inhibitor) were obtained from RiboBio (Guangzhou, China) and transfected with Lipofectamine 2000 (Invitrogen, United States).

### CircRNA Selection

For circRNAs selection, a circRNA chip (Capitalbiotech, Beijing, China) was used to predict the differentially expressed circRNAs. MiRanda was used to predict the potential binding relationship between miR-149-5p and circRNAs. circRNAs with potential miR-149-5p binding sites and significantly different expressions were selected for further verification. A larger sample size was used to verify the top three differentially expressed circRNAs (hsa_circ_0102690, hsa_circ_0120872, and hsa_circ_0027732) in the bone marrow of 30 ALL cases and 30 controls.

### RNA Isolation and Quantitative Real-Time PCR (qRT-PCR)

Total RNA was extracted from bone marrow and cells by TRIzol (Invitrogen, Carlsbad, CA, United States) based on the manufacturer’s protocol. We use HiScript Q RT SuperMix (Vazyme, Jiangsu, China) to reverse transcribe total RNA into cDNA for qPCR. Quantitative RT-PCR methods was performed with SYBR Green PCR Master Mix (Vazyme, Jiangsu, China). RNA relative expression was calculated by 2^–ΔΔCT^ method. GAPDH was used as internal control for quantification of circADD2 and AKT2, while U6 for miR-149-5p.

### CCK-8 Viability Assay

Cells transfected with overexpressed circADD2 were seeded into 96-well plates (5 × 10^3^ cells/well). After 0, 24, 48, or 72 h of incubation, 10 μL of CCK-8 (Dojindo, Kumamoto, Japan) was added into each well and allowed for incubation of 2 h. The solution was measured spectrophotometrically at 450 nm.

### Antibodies and Flow Cytometry Analysis

In cell apoptosis assay, the Annexin V-FITC/PI apoptosis detection kit (BD, United States) was used. First, the cells were seeded into six-well plates (5 × 10^5^ cells/well). After 48 h, the cells exposed to different treatments were collected and washed in cold phosphate buffered saline (PBS). Then, the cells were double-stained by 5 μL of propidium iodine (PI) and 5 μL of Annexin V-fluorescein isothiocyanate (FITC), at room temperature away from light for 15 min. The stained cells were detected by flow cytometer (Beckman Coulter, Brea, CA, United States).

The proportion of Ki-67 in cells was analyzed by flow cytometry to evaluate the protein levels of Ki-67. Then, the cells were seeded into six-well plates (5 × 10^5^cells/well). After 48 h, the cells exposed to different treatments were collected and washed for several times using PBS and 0.3 μL of phycoerythrin(PE)-conjugated mouse anti-Ki-67 antibody (Invitrogen, United States) was incubated with cells at room temperature for 20 min. Then the cells were detected by flow cytometer (Beckman Coulter, Brea, CA, United States), and the data were processed by CytExpert 2.0. We also detected Ki-67 value in untransfected ALL cells with stained antibody as isotype controls.

### Tumor Xenograft Assay

The study involved in mice model was permitted by the Animal Management Committee of Nanjing Medical University. A total of 5 × 10^7^ Jurkat cells steadily transfected with circADD2 or control vector were subcutaneously injected into 10 BALB/c nude mice (4–5 weeks old, male). Volume (V), length (L), and width (W) were measured every week and the tumors’ volume was calculated with the formula V = (W^2^ × L)/2. After 6 weeks, the mice were killed, the tumors were removed, and the weight of the tumor was measured.

### Nuclear and Cytoplasmic Extraction Assay

Nuclear and cytoplasmic fraction was separated, followed by RNA extraction. To isolate nuclear and cytoplasmic fractions, kit of Thermo Fisher (Invitrogen, AM1921, Lithuania) was applied according to the manufacturer’s agreement. The relative gene expression was calculated with 2^–ΔCT^ method.

### RNA Immunoprecipitation (RIP)

According to the manufacturer’s protocol, RIP was conducted by Magna RIP kit (Millipore, Billerica, MA, United States). Considering the low expression of endogenous miR-149-5p in 293T, we used miR-149-5p mimics to make our experiment results more prominent. 293T cells were then transfected with miR-149-5p mimics and NC mimics. RIP experiment was carried out using AGO2 antibody (anti-AGO2) (Millipore, Billerica, MA, United States). IgG was the negative control for AGO2, while miR-NC was the negative control for miR-149-5p mimics. After 48 h, the lysated cells were incubated with the RIP buffer containing magnetic beads conjugated to anti-AGO2 or control immunoglobulin G (anti-IgG) (Millipore, Billerica, MA, United States) overnight at 4°C overnight. After washing with buffer for several times, the qRT-PCR was performed to analyze the purified RNA.

### Dual-Luciferase Reporter Assay

For the dual-luciferase reporter assay, the wild-type and the mutated 3′UTRs of circADD2 containing the binding cites of miR-149-5p were subcloned into the pmiR-RB-Report^TM^ vectors (RiboBio, Guangzhou, China) to, respectively, get pmiR-RB-REPOR-circADD2-WT and pmiR-RB-REPORT-circADD2-MUT. The circADD2-WT primers were forward 5′-GCGCTCGAGTTTCCACCTGGATGTTTGAGGT-3′ and reverse 5′-AATGCGGCCGCTCATGGAAGATGTCGGGAAGA-3′, and the circADD2-MUT primers were forward 5′-ACC CCCACCTCTGTGACGAGTGAACAGAACCTCGGTTCCTCA GGGCTGGGCCAGCCTCC-3′ and reverse 5′-GAGG AACCGAGGTTCTGTTCACTCGTCACAGAGGTGGGGGTA GCTCCACTCTCAAGGGTGC-3′. MiR-149-5p mimics and NC mimics were cotransfected into 293T cells, with the luciferase reporter vectors, respectively. Luciferase activity was measured 48 h later according to manufacture’s procedures (Promega, Madison, WI, United States).

### Western Blot Analysis

The total protein of cells was exacted with RIPA buffer, separated with SDS-PAGE and transferred onto PVDF membranes (Bio-Rad, CA, United States). The membranes were blocked with 5% skimmed milk powder and incubated with primary antibodies against AKT2, p-AKT2, p-AKT, AKT (CST, 1:1,000) and GAPDH (CST, 1:2,000) at 4°C overnight, then incubated with secondary antibodies (CST, 1:5,000) at room temperature for 1 h. Membranes were detected by chemiluminescence system(Bio-Rad).

### Immunohistochemistry (IHC)

As for IHC staining, after procedures of dewaxing, rehydration and antigen retrieval, the sections were incubated with primary anti-AKT2 antibody (CST, 1:1,000) overnight at 4°C, then with secondary antibody for 2 h at 37°C. The slices were added with HRP-labeled streptavidin solution, then stained by 3,30-diaminobenzidine (DAB), counterstained with hematoxylin, and photographed under a microscope (Leica, Wetzlar, Germany).

### Bioinformatics Analyses

We used several databases to pinpoint the target genes of miR-149-5p. miRTarBase^1^, miRWalk^2^, and StarBase^3^. The miR-149-5p-mRNAs regulatory networks were visualized using the Cystoscope software V3.8.0.

### Statistical Analysis

All data were presented as the means ± standard error of mean (SEM). Unpaired *t*-tests was used to compare group differences. Statistical analyses were conducted using GraphPad Prism 8 software (GraphPad Prism, Inc., La Jolla, CA, United States). *P* < 0.05 was considered significant.

## Results

### Selection of CircADD2 in Childhood ALL

**FIGURE 1:**
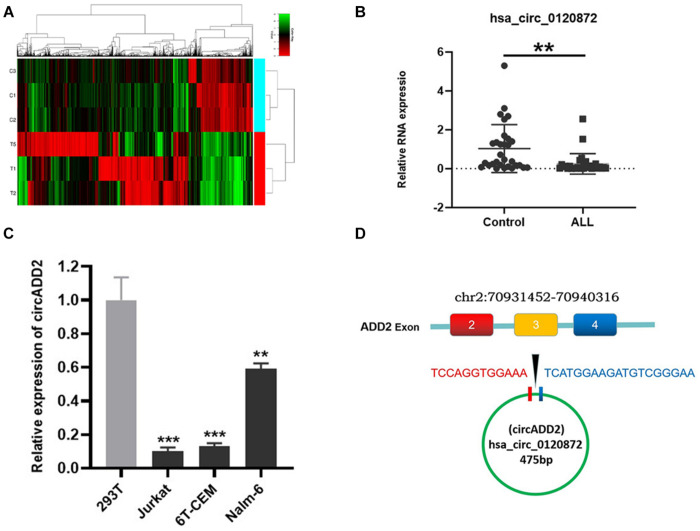
Detection of circRNA expression in childhood ALL. **(A)** A heatmap showing the differentially expressed circRNAs in ALL and control groups. The results showed a distinguishable circRNA expression profile among samples. Red: upregulation; green: downregulation; black: no change. **(B)** qRT-PCR confirmed the low expression of circADD2 in children with ALL compared with control group (*n* = 30). **(C)** The circADD2 expression in ALL cell lines was prominently lower than the normal cell line 293T. **(D)** Genomic loci of ADD2 and circADD2. Data were showed as mean ± SD and analyzed by unpaired *t*-test, **P < 0.01, ***P < 0.001.

We used a chip to detect the differentially expressed circRNAs in the bone marrow of children with ALL and the non-tumor control group (Figure 1A). We have uploaded the raw data and analyzed data to a public database, which could be checked in https://www.ncbi.nlm.nih.gov/geo/query/acc.cgi?acc=GSE166579. Based on the cutoff value of corrected *P*-value < 0.05, we selected 11163 circRNAs with differential expression, including 3976 up-regulated and 7187 down-regulated. To determine the circRNAs regulating miR-149-5p, we calculated the sequence matching and free energy between circRNAs and miRNAs based on the miRanda algorithm. Then we selected circRNAs with potential miR-149-5p binding sites and significantly differential expressions between ALL and non-ALL tissues (*p* < 0.05). We continued to expand the sample size to verify the top three differentially expressed circRNAs (hsa_circ_0102690, hsa_circ_0120872, and hsa_circ_0027732) in the bone marrow of 30 ALL cases and 30 controls. The results showed that the expression level of hsa_circ_0120872 in children with ALL was significantly lower in the control group, which was consistent with the results of preliminary screening (Figure 1B). Besides, has_circ_0120872 was found lowly expressed in ALL cells (Jurkar, 6T-CEM and Nalm-6) than in normal cell line 293T (Figure 1C). Hsa_circ_0120872 was derived from exon 2-4 of ADD2 gene located in the region chr2:70931452-70940316. The ADD2 gene sequence was 8,864 bp in length and the spliced mature circRNA was 475 bp (Figure 1D). We therefore named hsa_circ_0120872 as circADD2.

### CircADD2 Inhibits the Proliferation of ALL Cells *in vitro*

**FIGURE 2:**
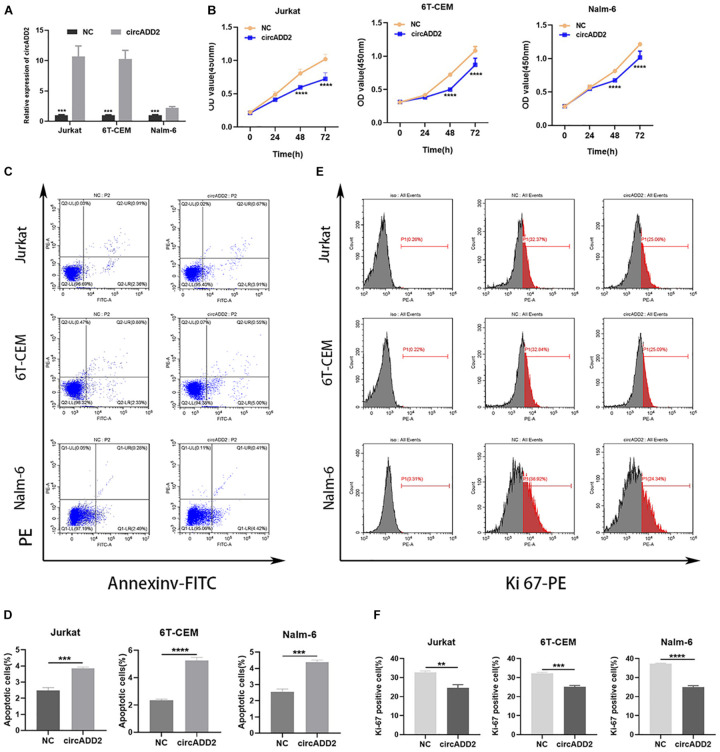
CircADD2 overexpression suppressed ALL progression. **(A)** Expression of circADD2 was detected in Jurkat, 6T-CEM and Nalm-6 cells transfected with circADD2 overexpression vector and control vector by qRT-PCR. Results indicated successful overexpression of circADD2. **(B)** CCK-8 assay showed that after circADD2 overexpressing vector was transfected in Jurkat, 6T-CEM and Nalm-6 cells for 48 and 72 h, proliferation of the ALL cells was significantly inhibited. **(C,D)** Apoptotic rate of Jurkat, 6T-CEM and Nalm-6 cell lines was analyzed by flow cytometry. After overexpression of circADD2, ALL cell apoptotic rate was remarkably increased. **(E,F)** The ratio of Ki-67 in Jurkat, 6T-CEM and Nalm-6 cells was detected by flow cytometry. The level of Ki-67 positive cells was significantly reduced after overexpression of circADD2. Data were showed as mean ± SD and analyzed by unpaired *t*-test, **P < 0.01, ***P < 0.001, ****p < 0.0001.

To explore the biological function of circADD2 in ALL, we overexpressed circADD2 in ALL cell lines (Figure 2A). CCK-8 assay demonstrated that circADD2 overexpression significantly decreased the proliferation of Jurkat, 6T-CEM and Nalm-6 cells after a transfection of 48 h and 72 h, as shown by their growth curves (Figure 2B). As shown in Figures 2C,D, compared with NC groups, the proportion of apoptotic ALL cells in circADD2 overexpressed groups were significantly elevated, revealing that circADD2 overexpression promoted cell apoptosis. Meanwhile, we detected the level of Ki-67, a typical marker of proliferation. Lower level of Ki-67 was observed in ALL cells with overexpressed circADD2 (Figures 2E,F), which was also consistent with the results of CCK-8 assay.

### CircADD2 Suppresses Tumor Growth *in vivo*

**FIGURE 3:**
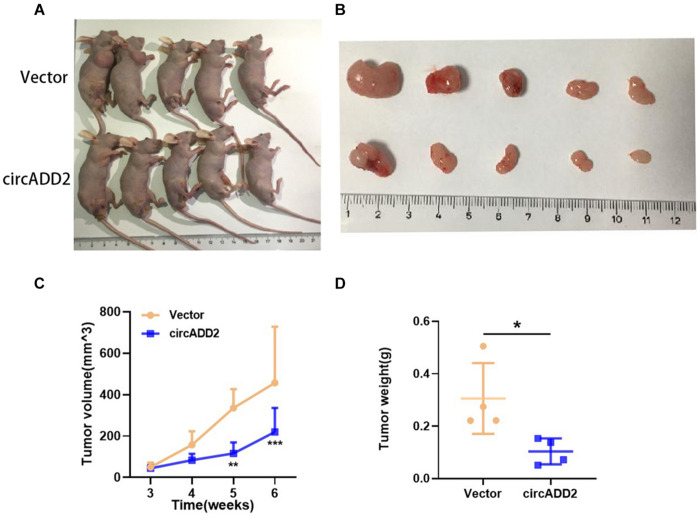
Over-expressed circADD2 Jurkat cells were injected into nude mice to detect the effect of circADD2 (*n* = 5) *in vivo*. **(A)** Jurkat cells stably transfected with circADD2 or control vector were injected into nude mice with established subcutaneous xenograft tumors. **(B)** Representative pictures of xenograft tumor formation in nude mice (top: control group; bottom: treated group). **(C,D)** Compared with the vector group, circADD2-treated nude mice exhibited significantly smaller tumor volume and significantly lighter tumor weight after cell injection for 5–6 weeks. Data were showed as mean ± SD and analyzed by unpaired *t*-test, *P < 0.05, **P < 0.01, ***P < 0.001.

Additionally, to investigate whether circADD2 overexpression retards ALL growth *in vivo*, a subcutaneous tumor mice model was constructed. Jurkat cells stably transfected with circADD2 or control vector were injected subcutaneously into the nude mice. Three weeks after injection, the tumor volumes were measured weekly. The results showed that circADD2 overexpression significantly inhibited tumor growth *in vivo* (Figures 3A,B). As shown in Figure 3C, tumor growth curves were made in different groups. The tumor volume was significantly reduced in circADD2 overexpressed group 5–6 weeks after cell injection. Then, we removed the tumor tissue and weighed it. Compared with the control group, the weight of tumor was significantly lighter in circADD2-treated group (Figure 3D). Taken together, circADD2 suppressed ALL progression, both *in vitro* and *in vivo*.

### CircADD2 May Regulate Cell Proliferation and Apoptosis Through Sponging miR-149-5p

**FIGURE 4:**
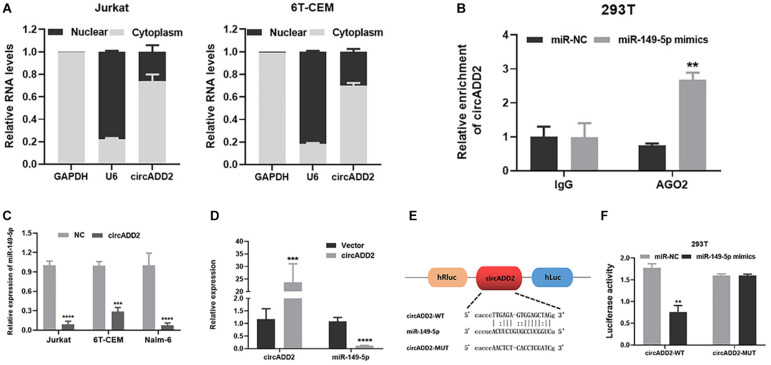
CircADD2 sponged miR-149-5p. **(A)** Cellular localization of circADD2 in Jurkat and 6T-CEM. Results revealed that circADD2 was mainly expressed in the cytoplasm ALL cells. **(B)** CircADD2 levels in purified RNA collected from products of anti-AGO2 or anti-IgG was detected by RT-qPCR. After miR-149-5p mimics was transfected into 293T cells, significant enrichment of circADD2 in the RNA complex immunoprecipitated by AGO2 antibody was observed. **(C)** qRT-PCR confirmed the decreased level of miR-149-5p in ALL cells after overexpression of circADD2. **(D)** CircADD2 and miR-149-5p levels were detected by qRT-PCR in tumor cells from xenograft generated by Jurkat cells stably transfected with circADD2 or control vector. MiR-149-5p level was significantly decreased after circADD2 overexpression *in vivo*, while circADD2 level was elevated. **(E,F)** Dual luciferase reporter assay was used to prove the binding between circADD2 and miR-149-5p. The fluorescence activity of circADD2–WT was reduced and the fluorescence activity in circADD2–MUT was basically unchanged when transfected with miR–149-5p mimics. Data were showed as mean ± SD and analyzed by unpaired *t*-test, **P < 0.01, ***P < 0.001, ****P < 0.0001.

To investigate the enrichment of circADD2 in the cells, we extracted and separated cytoplasmic RNA and nuclear RNA and evaluated circADD2 expression using qRT-PCR. Results revealed that circADD2 was preferentially expressed in the cytoplasm of Jurkat and 6T-CEM cells (Figure 4A). Given that circADD2 was enriched in the cytoplasm, we then carried out RNA immunoprecipitation assay (RIP) with argonaute2 (AGO2) antibodies. Studies have shown that circRNA-AGO2-miRNA may form a ternary complex. To increase the percentage of the miR-149-5p-AGO2 complex, miR-149-5p mimics were transfected into 293T cells, the expression of miR-149-5p increased 12.05-fold (Supplementary Figure 2), and then we detected significant enrichment of circADD2 in the RNA complex immunoprecipitated by AGO2 antibody, indicating the potential combination of miR-149-5p with circADD2 (Figure 4B). Furthermore, we found that overexpression of circADD2 reduced miR-149-5p level in ALL cells (Figure 4C). We also detected the expression levels of miR-149-5p and circADD2 in RNA extracted from the tissue of mouse tumor that was induced by subcutaneously injected Jurkat cells. Results suggested that miR-149-5p level was significantly decreased after circADD2 overexpression *in vivo*, while circADD2 level was elevated (Figure 4D). Then, the luciferase reporter assay was used to demonstrate the interaction between circADD2 and miR-149-5p in 293T cells, and the results showed that miR-149-5p significantly reduced the luciferase activity of circADD2 WT reporter, but not that of the reporters with mutated miR-149-5p binding site (Figures 4E,F). These results suggest that circADD2 sponges miR-149-5p.

**FIGURE 5:**
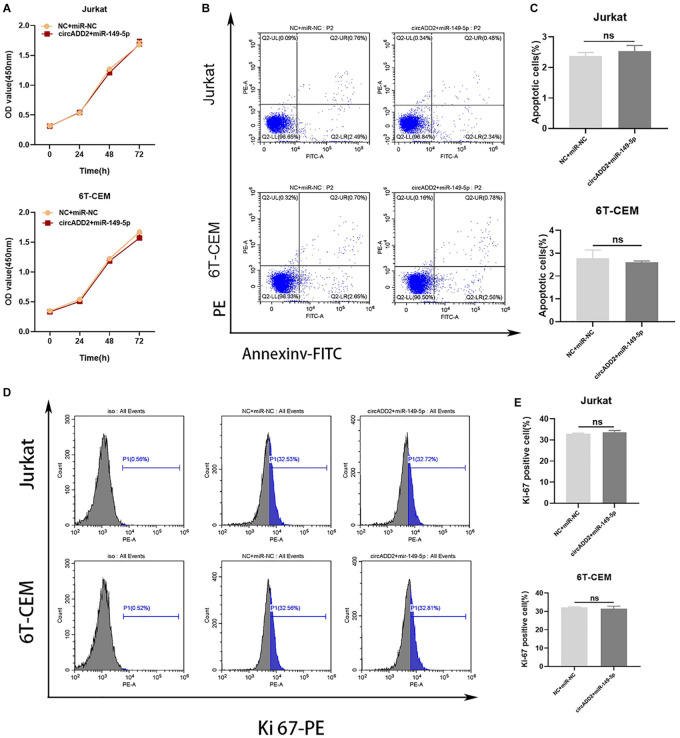
MiR-149-5p overexpression eliminated the effect of circADD2 overexpression. **(A)** CCK-8 assay showed no significant change in proliferation rate after circADD2 overexpressing plasmid and miR-149-5p were co-transfected into Jurkat and 6T-CEM cells. **(B,C)** Apoptotic rate of cells transfected with NC + miR-NC or circADD2 + miR-149-5p was analyzed by flow cytometry. Compared with the NC + miR-NC groups, there was no change in apoptosis in the circADD2 + miR-149-5p groups. **(D,E)** There was no remarkable difference of the levels of Ki-67-positive cells between circADD2 + miR-149-5p group and NC + miR-NC group. Data were showed as mean ± SD and analyzed by unpaired *t*-test, ns: no significance.

To confirm whether circADD2 acts by sponging miR-149-5p, circADD2 overexpressing plasmid and miR-149-5p were co-transfected into Jurkat and 6T-CEM cells in rescue assays. The cells transfected with NC plus miR-NC were used as controls. The proliferative rate showed no significant change after circADD2 overexpressing plasmid and miR-149-5p were co-transfected into Jurkat and 6T-CEM cells (Figure 5A). We observed that compared with the NC + miR-NC groups, the apoptosis did not change in circADD2 + miR-149-5p groups (Figures 5B,C). Also, there was no remarkable difference in the levels of Ki-67-positive cells between treated and control groups (Figures 5D,E). Taken together, circADD2 sponged miR-149-5p, with competition in their regulation.

### CircADD2 Modulates the Expression of AKT2

**FIGURE 6:**
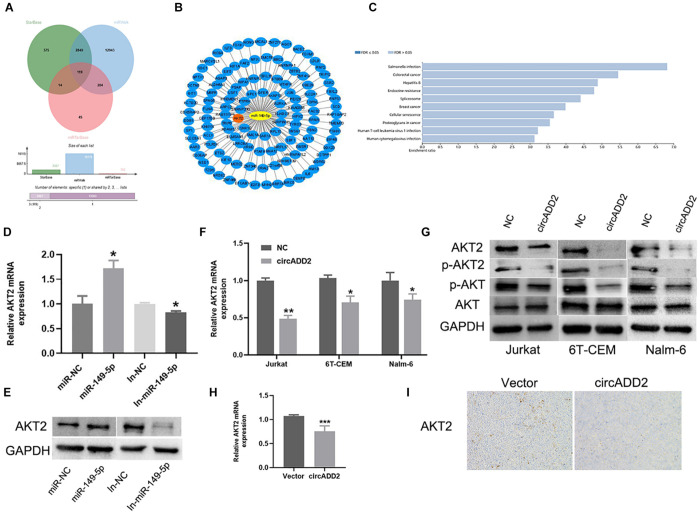
Overexpression of circADD2 reduced the expression of AKT2. **(A)** The Venn plots showed downstream genes binding to miR-149-5p. **(B)** Cytoscape graphs of miR-149-5p and target genes**. (C)** KEGG enriched the signal pathways of miR-149-5p target genes. **(D,E)** Jurkat cells transfected with miR-149-5p mimics or miR-149-5p inhibitor. The level of AKT2 was detected by qRT-PCR and western blot. The results showed that miR-149-5p increased AKT2 expression, while inhibition of miR-149-5p decreased AKT2 expression. **(F,G)** Relative expression of AKT2 was detected by qRT-PCR and Western blot in ALL cells transfected with circADD2. We found that after overexpression of circADD2, the level of AKT2 and its phosphorylation decreased. **(H)** AKT2 levels were detected by qRT-PCR in tumor cells from xenograft generated by Jurkat cells stably transfected with circADD2 or control vector. AKT2 level was significantly decreased after circADD2 overexpression *in vivo*. **(I)** IHC staining was used to detect the expression of AKT2 in the xenografts, and the level of AKT2 in the circADD2 groups was significantly reduced. Data were showed as mean ± SD and analyzed by unpaired *t*-test, *P < 0.05, **P < 0.01, ***P < 0.001.

We exported the downstream genes of miR-149-5p through databases (i.e., miRTarBase, miRWalk, and StarBase), finding that 119 mRNAs were overlapped (Figure 6A). We used Cytoscape to visualize the 119 target genes of miR-149-5p (Figure 6B). Using Kyoto Encyclopedia of Genes and Genomes (KEGG) pathway analysis, the enrichment pathways of these 119 target genes are shown in Figure 6C. We found that AKT2 was highly enriched in these signaling pathways. Interestingly, AKT2 was a gene predicted in all miRTarBase, miRWalk, and StarBase databases. Then, we selected AKT2 as one target gene of miR-149-5p. Western blot and qRT-PCR results showed that overexpression of miR-149-5p increased the level of AKT2, and inhibition of miR-149-5p reduced the level of AKT2 (Figures 6D,E). Considering the function of ceRNA, we hypothesized that cirADD2 may regulate the expression of AKT2 through sponging miR-149-5p. As expected, we found that circADD2 overexpression markedly decreased the protein and mRNA levels of AKT2 in Jurkat, 6T-CEM and Nalm-6 cells, as well as the protein level of p-AKT2 (Figures 6F,G). Moreover, we removed the animal tumors and extracted RNA. The results showed that circADD2 overexpression reduced the expression of AKT2 *in vivo* (Figure 6H). IHC (Immunohistochemistry) analysis showed that the expression of AKT2 was reduced in the tumors formed by ALL cells over-expressing circADD2 (Figure 6I). These results suggested that circADD2 could regulate the expression of AKT2 in ALL.

## Discussion

CircRNAs, as a class of abundant and stable endogenous ncRNAs, are gaining considerable attention for their potentials of regulating cancer development (Jeck and Sharpless, 2014; Geng et al., 2018). Our study reported the down-regulation of circADD2 in childhood ALL bone marrow and ALL cell lines. Our *in vitro* and *in vivo* experiments verified that circADD2 could competitively bind to miR-149-5p to adversely affected ALL cell lines *in vitro* and *in vivo*, reducing AKT2 expression, and thereby inhibit the progression of ALL.

Studies have shown that circRNAs are differentially expressed between tumor and normal tissues, suggesting their important roles in tumorigenesis (Song et al., 2016; Lu et al., 2018). In the present study, we first identified the differentially expressed circRNAs in childhood ALL through microarray analysis. Next, we screened out circADD2, a significantly down-regulated circRNA with a potential miR-149-5p binding site. In the present study, we confirmed the low expression of circADD2 in bone marrow samples of 30 children with ALL, as well as three ALL cell lines. Subsequently, the functional analysis showed that the overexpression of circADD2 in Jurkat, 6T-CEM and Nalm-6 cells could inhibit the proliferation and apoptosis of ALL cells. These results suggested that circADD2 overexpression could suppress the proliferation and promote apoptosis of ALL cells.

Accumulating evidence indicates that circRNAs may function through competitively binding to miRNAs (ceRNAs), and the ceRNA hypothesis indicates that circRNAs may sponge miRNA to counter the latter’s effects on its target genes (Zhong et al., 2018; Chen S. et al., 2019). For example, circCCDC9 sponges miR-6792-3p to suppress the progression of gastric cancer through up regulating CAV1 expression (Luo et al., 2020). In another example, circCTNNA1 promotes colorectal cancer progression via circCTNNA1/miR-149-5p/FOXM1 axis (Chen et al., 2020). Also, circSLC8A1 plays a suppressive role in bladder cancer progression via sponging miR-130b/miR-494 and upregulating downstream PTEN (Lu et al., 2019). It should be noted that miR-149-5p has been identified as an oncogene in childhood leukemia. For instance, Tian demonstrated that miR-149-5p was highly expressed in AML, and could promote AML progression by decreasing cell apoptosis (Tian and Yan, 2016). Another example showed miR-149-5p was highly expressed in T-ALL, and that miR-149-5p functioned as an oncogene via regulating proliferation, cell cycle, and apoptosis (Fan et al., 2016). Based on these observations, we aimed to identify how circADD2 works in childhood ALL through sponging miR-149-5p.

AGO2 is an indicator protein in the sponging of circRNA, and studies have confirmed that circRNA-AGO2-miRNA may form a ternary complex (Chen Y. et al., 2019). In the present study, the RIP assay of 293T cells confirmed that circADD2 could bind to AGO2 and act as miRNAs sponge. Using dual-luciferase reporter assay, the present study revealed that circADD2 could sponge miR-149-5p in ALL cells, laying a foundation for further research on the biological characteristics of circADD2. This evidence proved the potential of circADD2 as a novel biomarker and a therapeutic target in childhood ALL.

Furthermore, we used some bioinformatics tools to filter out the target genes of miR-149-5p. Accordingly, KEGG analysis revealed that AKT2 was significantly enriched in several cancer-related signaling pathways, suggesting that AKT2 may act as a cancer gene in childhood ALL. Studies have also demonstrated that AKT2 plays important roles in the carcinogenesis of leukemia (Gong et al., 2014; Ying et al., 2018). Most studies have shown that miRNAs could repress the expression of target genes by binding to the 3′untranslated region (3′UTR) (Hausser et al., 2013; Wang et al., 2015). However, our study discovered that miR-149-5p overexpression up-regulated AKT2 in ALL cells. We inferred that after miR-149-5p is overexpressed, AKT2 was upregulated in a compensatory manner. Besides, although many studies have suggested that miRNA can down-regulate its target genes, miRNA can play positive roles in regulating the expression of target genes. miRNAs positively regulate gene transcription through targeted promoters, a process called RNA activation (RNAa) (Huang et al., 2012; Zheng et al., 2014; Portnoy et al., 2016). A subset of miRNAs from enhancer sites can activate transcription and function as an activator (Xiao et al., 2017). This may explain our findings. In the present study, we found that circADD2 could reduce the expression of AKT2, a condition probably involving ceRNA mechanism of circADD2 and miR-149-5p. Considering the promotive role of AKT2 in leukemia, we hypothesized that circADD2 may sponge miR-149-5p to regulate AKT2 expression in childhood ALL. Further studies should be conducted to confirm AKT2 as the direct effector of circADD2/miR-149-5p/AKT2 axis in the pathogenesis of ALL.

Here we identified that circADD2 could restrain ALL progression. Further studies with a larger sample size, including bone marrow samples collected in both diagnosis and treatment process of childhood ALL, should be organized to comprehensively evaluate the value of circADD2 as a biomarker in childhood ALL.

## Conclusion

The expression of circADD2 is down-regulated in childhood ALL. This circRNA can sponge miR-149-5p to inhibit the proliferation of leukemia cells *in vitro* and *in vivo*. circADD2 can regulate the expression of AKT2 probably through its ceRNA effect on miR-149-5p. circADD2 may serve as a potential biomarker and a therapeutic target in childhood ALL.

## Data Availability Statement

The datasets presented in this study can be found in online repositories. The names of the repository/repositories and accession number(s) can be found below: NCBI Gene Expression Omnibus, accession no: GSE166579.

## Ethics Statement

The studies involving human participants were reviewed and approved by the Ethics Committee of Nanjing Medical University. Written informed consent to participate in this study was provided by the participants’ legal guardian/next of kin. The animal study was reviewed and approved by Animal Management Committee of Nanjing Medical University. Written informed consent was obtained from the minor(s)’ legal guardian/next of kin for the publication of any potentially identifiable images or data included in this article.

## Author Contributions

YF, YX, and YZ contributed to the design of the study. YZ, XM, and HZ wrote the first edition of the manuscript and performed the experiments. YW and MK made the figures. YF and YX revised the version of the manuscript finally. All authors have reviewed and approved this manuscript and consented to publish this manuscript.

## Conflict of Interest

The authors declare that the research was conducted in the absence of any commercial or financial relationships that could be construed as a potential conflict of interest.

## Footnotes

1 http://mirtarbase.cuhk.edu.cn/php/index.php

2 http://mirwalk.umm.uni-heidelberg.de/

3 http://starbase.sysu.edu.cn/
