# Supplementary figures and images for: Mechanism of circADD2 as ceRNA in Childhood Acute Lymphoblastic Leukemia
Source: Front Cell Dev Biol. 2021 May 13;9:639910. doi: 10.3389/fcell.2021.639910 (PMC8155473; doi:10.3389/fcell.2021.639910)

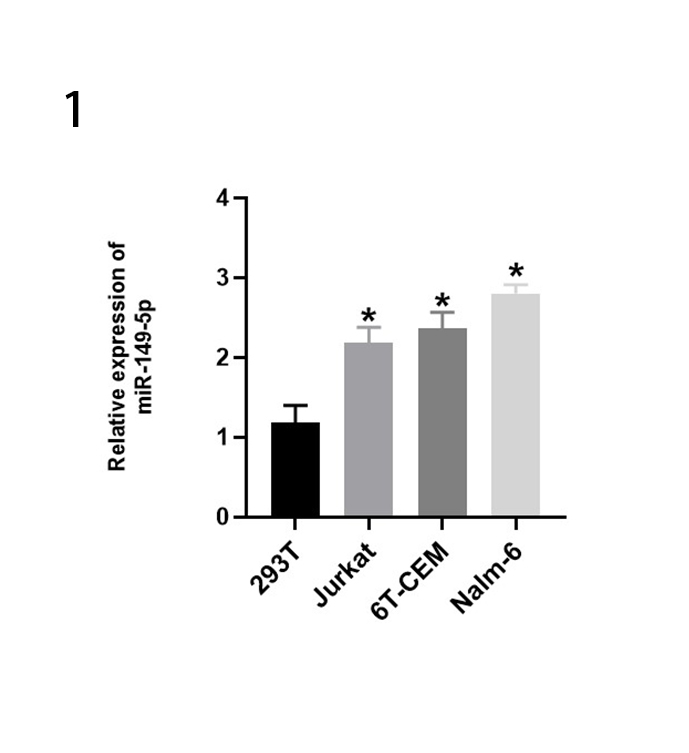

Supplement: Supplementary file 1 [file Image_1.JPEG]

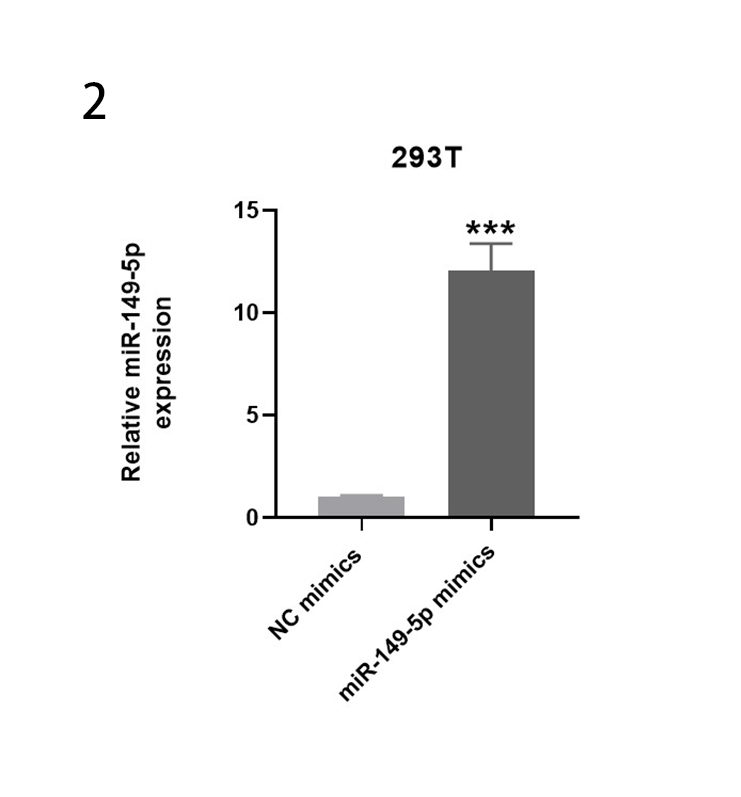

Supplement: Supplementary file 2 [file Image_2.JPEG]
